# Supplementary material for: Siglec-9 defines and restrains a natural killer subpopulation highly cytotoxic to HIV-infected cells
Source: PLoS Pathog. 2021 Nov 11;17(11):e1010034. doi: 10.1371/journal.ppat.1010034 (PMC8584986; doi:10.1371/journal.ppat.1010034)
Supplement: S1 Table — (DOCX) [file ppat.1010034.s012.docx]

**S1 Table.** Clinical data of the study participants whose cells were used for the experiments in Figs 1, 2, and 3A.

| **Donor ID** | **HIV**  **status** | **Age**  **(years)** | **Gender** | **CD4 count (cells/mm^3^)** | **VL (copies/ml)** |
| --- | --- | --- | --- | --- | --- |
| **NC01** | Negative | 42 | Male | - | - |
| **NC02** | Negative | NA | Female | - | - |
| **NC03** | Negative | 43 | Male | - | - |
| **NC04** | Negative | NA | Male | - | - |
| **NC05** | Negative | NA | NA | - | - |
| **NC06** | Negative | 57 | Female | - | - |
| **NC07** | Negative | NA | NA | - | - |
| **NC08** | Negative | 32 | Male | - | - |
| **NC09** | Negative | 39 | Male | - | - |
| **NC10** | Negative | 32 | Male | - | - |
| **ART01** | Positive ART-suppressed | 31 | Male | 546 | < 20 |
| **ART02** | Positive ART-suppressed | 40 | Male | 926 | <20 |
| **ART03** | Positive ART-suppressed | 38 | Male | 940 | < 20 |
| **ART04** | Positive ART-suppressed | 48 | Male | 763 | < 20 |
| **ART05** | Positive ART-suppressed | 34 | Male | 1055 | 112 |
| **ART06** | Positive ART-suppressed | 61 | Male | 475 | < 20 |
| **ART07** | Positive ART-suppressed | 29 | Male | 516 | 24 |
| **ART08** | Positive ART-suppressed | 30 | Male | 1195 | < 20 |
| **ART09** | Positive ART-suppressed | 43 | Male | 508 | < 20 |
| **ART10** | Positive ART-suppressed | 61 | Male | 766 | <20 |
| **VIR01** | Positive Viremic | NA | NA | 470 | 34,286 |
| **VIR02** | Positive Viremic | NA | NA | 780 | 13,948 |
| **VIR03** | Positive Viremic | NA | NA | 210 | 400,000 |
| **VIR04** | Positive Viremic | NA | NA | 560 | 19,992 |
| **VIR05** | Positive Viremic | NA | NA | 220 | 1,216,914 |
| **VIR06** | Positive Viremic | NA | NA | 320 | 25,947 |
| **VIR07** | Positive Viremic | 25 | Male | 364 | 711,588 |
| **VIR08** | Positive Viremic | 24 | Male | 453 | 6,907,692 |
| **VIR09** | Positive Viremic | 26 | Male | 410 | 1,692,571 |
| **VIR10** | Positive Viremic | 22 | Male | 547 | 47,228 |
| **VIR11** | Positive Viremic | 33 | Male | 697 | 201,241 |

NA = not available
