# Supplementary material for: Siglec-9 defines and restrains a natural killer subpopulation highly cytotoxic to HIV-infected cells
Source: PLoS Pathog. 2021 Nov 11;17(11):e1010034. doi: 10.1371/journal.ppat.1010034 (PMC8584986; doi:10.1371/journal.ppat.1010034)
Supplement: S2 Table — (DOCX) [file ppat.1010034.s013.docx]

**S2 Table.** Clinical data of the study participants whose cells were used for the experiments in Fig 3B.

| **Donor ID** | **HIV**  **status** | **Age**  **(years)** | **Gender** | **CD4 count (cells/mm^3^)** | **VL (copies/ml)** |
| --- | --- | --- | --- | --- | --- |
| **ART11** | Positive ART-suppressed | 72 | Male | 336 | <50 |
| **ART12** | Positive ART-suppressed | 54 | Male | 491 | <50 |
| **ART13** | Positive ART-suppressed | 65 | Male | 462 | <50 |
| **ART14** | Positive ART-suppressed | 65 | Male | 822 | <50 |
| **ART15** | Positive ART-suppressed | 53 | Male | 533 | <50 |
| **ART16** | Positive ART-suppressed | 52 | Male | 659 | <50 |
| **ART17** | Positive ART-suppressed | 48 | Male | 486 | <50 |
| **ART18** | Positive ART-suppressed | 55 | Female | 632 | <50 |
| **ART19** | Positive ART-suppressed | 52 | Male | 574 | <50 |
| **ART20** | Positive ART-suppressed | 65 | Male | 987 | <50 |
| **ART21** | Positive ART-suppressed | 72 | Male | 713 | <50 |
