# Supplementary material for: Siglec-9 defines and restrains a natural killer subpopulation highly cytotoxic to HIV-infected cells
Source: PLoS Pathog. 2021 Nov 11;17(11):e1010034. doi: 10.1371/journal.ppat.1010034 (PMC8584986; doi:10.1371/journal.ppat.1010034)
Supplement: Attachment — Submitted filename: Response_V6_091721.pdf [file ppat.1010034.s014.pdf]

THE WISTAR INSTITUTE

MOHAMED ABDEL-MOHSEN, PH.D.
Assistant Professor
The Wistar Institute
3601 Spruce Street
Philadelphia, PA 19104
P (215) 898-3968 E mmohsen@Wistar.org

Point-by-point responses

We are grateful to the reviewers for providing us with very helpful and constructive feedback, which has significantly improved our manuscript. Our responses to the reviewers' comments are in blue text to facilitate the review process. We have included two versions of the revised manuscript; one with changes highlighted and one with changes accepted.

We have performed several new experiments and made several modifications throughout the manuscript to address the reviewers' concerns. A partial list of the new experimental results is presented here:

- Using autologous NK cell killing assays, we showed that Siglec-9^{+} CD56^{dim} NK cells are more cytotoxic against autologous HIV-infected primary CD4^{+} T cells than are Siglec-9^{-} CD56^{dim} NK cells (this experiment was independently repeated three times using cells from three donors, and each repeat was done in multiple technical replicates: **new Figure 4E-F**).
- Using autologous NK cell killing assays, we showed that the Siglec-9 blocking antibody selectively induces the cytotoxicity of Siglec-9^{+} CD56^{dim} NK cells against autologous HIV-infected primary CD4^{+} T cells (this experiment was independently repeated three times using cells from three donors, and each repeat was done in multiple technical replicates: **new Figure 5D-F** and **new Supplementary Figure 7**).
- Using Fc receptor blocker, as suggested by the reviewers, we showed that the HIV broadly neutralizing antibodies (bNAb)-sialidase conjugate directly enhances NK cytotoxic responses against HIV-infected targets (**new Figure 7G**).
- Using autologous killing assays, we demonstrate that the bNAb-Sialidase conjugate enhances NK cytotoxicity against autologous HIV-infected primary CD4^{+} T cells compared to bNAb only control (these experiments were independently repeated three times using cells from three donors, and each repeat was done in multiple technical replicates: **new Figures 8 and 9**).
- We re-analyzed the flow-cytometry data in Figures 1 and 2 to examine the effects of including HLA-DR^{+} and CD7^{+} cells on our findings (**new Supplementary Figures 2 and 3**), based on the reviewers' recommendations.
- Using NK cells from HIV-infected ART-suppressed individuals and CEM.NKR cells (the experiment was repeated twice with 3-4 replicates each), we were able (using live cell imaging) to show that the bNAb-sialidase conjugate enhances the ability of NK cells from HIV+ individuals to kill HIV-infected cells with minimal impact on the HIV-negative cells (**new Figure 7H-I** and **new Supplementary Figure 10**).

Reviewer #1

Reviewer general comment: In this study, Adeniji et al. evaluate the expression of Siglec-9, a lectin with inhibitory functions, on natural killer cells in the context of HIV-1 infection. They showed that Siglec-9+ NK cells are depleted from PBMCs of HIV-1 infected individuals (both in viraemic and ART-treated patients) as compared to healthy individuals, which has not been reported previously. Using high dimensional flow cytometry, the authors show that during HIV-1 infection, Siglec-9+ NK cells displayed higher expression of activated receptors and lower of inhibitory ones as compared to their Siglec-9neg counterpart. Interestingly, the authors also observed an inverse correlation between Siglec-9 expression of NK cells and HIV-1 DNA levels in CD4 T cells from ART-treated HIV-1 patients, similarly of what has been reported during HBV infection (PMID: 29899741). Evaluating the functions of Siglec-9+ cells against HIV-1 infected cell lines, they convincingly demonstrate (using various assays and different cell lines) that Siglec-9+ NK cells from healthy

THE WISTAR INSTITUTE

MOHAMED ABDEL-MOHSEN, PH.D.
Assistant Professor
The Wistar Institute
3601 Spruce Street
Philadelphia, PA 19104
P (215) 898-3968 E mmohsen@Wistar.org

donors have higher cytotoxic capacities than Siglec-9neg NK cells. Those cytotoxic functions were enhanced by an anti-Siglec-9 blocking antibody.

The authors further develop and make use of Sialidase coupled to HIV-1 broadly neutralizing antibodies (bNAbs) (bNAb-STSia conjugates) as a proof-of-concept to demonstrate that those constructs are more potent than bNAbs alone in enhancing NK killing of HIV-1 infected cell lines.

Those interesting results are novel and may open new ways aiming at a 'functional cure' against HIV-1 infection. However, the reported decrease in Siglec-9+ NK cells in HIV-1 patients together with the absence of data on the effect of the bNAb-STSia conjugates on NK cells from HIV-1 infected patients limit the significance of the study.

Authors response: We appreciate the reviewer's opinion of our findings and that he/she found them novel. As detailed below, we have now addressed the concerns raised (above, and in the "Major comments" below), with multiple new experiments and modifications throughout the manuscript.

Major comments:

1. *The main limitation of the study is the use of NK cells isolated from healthy donors in their in vitro killing assays against HIV-1 infected cell lines. As pointed by the author (line 289), it would be important to validate the effect of the bNAb-STSia conjugates observed on HIV-1 infected cell lines using autologous HIV-1 infected CD4+ T cells.*

   Authors response: We thank the reviewer for this valuable suggestion. We have performed several new experiments using autologous NK and HIV-infected primary cells to support many of our original findings. First, in the **new Figure 4E-F**, we show that Siglec-9^{+} CD56^{dim} NK cells are more cytotoxic against autologous HIV-infected CD4^{+} T cells than are Siglec-9^{-} CD56^{dim} NK cells from the same donors. Second, we show that Siglec-9^{+} blocking antibody selectively increases Siglec-9^{+} CD56^{dim} NK degranulation (**new Figure 5D-E**) and decreases levels of HIV infection (**new Figure 5D, F and new Supplementary Figure 7**) in autologous HIV-infected CD4^{+} T cells. Finally, we show that the bNAb-Sialidase conjugate increases NK degranulation (**new Figure 8**) and decreases HIV infection (**new Figure 9**) in an autologous killing assay compared to the bNAb alone.

   Each of these experiments was repeated independently three times using cells from different donors, and each repeat was done in multiple technical replicates.

   We again thank the reviewer for raising this critical point, as we believe the addition of these new autologous experiments added more depth and sophistication to our manuscript.

2. *The use of NK cells isolated from HIV-1 infected individuals in these setting would also be important, especially as the authors report that Siglec-9+ NK cells are depleted in those individuals, thus possibly limiting the effect of the bNAb-STSia conjugates.*

   Authors response: We thank the reviewer for this suggestion; however, each of these autologous assays requires hundreds of millions of PBMCs (to ensure the isolation of enough CD4^{+} T cells and/or NK cells) and requires the usage of freshly isolated NK cells (as cryopreservation can impair NK cytotoxicity; Mark et al., *Nature Communications*, 2020 PMID: 33067467). Acquiring this large number of freshly isolated PBMCs (usually ~300 ml of blood) from several HIV-infected ART-suppressed individuals is challenging. As described above, we therefore took the approach of performing several autologous killing experiments using primary cells from HIV-uninfected individuals to support our original findings.

   In addition, we used cells from HIV-infected ART-suppressed individuals (this experiment was repeated twice using cells from donors ART09 and ART05; each with 3-4 replicates) to demonstrate that the bNAb-sialidase conjugate can enhance NK cells' ability to selectively eliminate HIV-infected cells without significantly impacting HIV-negative cells (using live cell imaging). This was done by mixing uninfected CEM.NKR cells after labeling them with PKH26 Red (red dye) with HIV-infected

MOHAMED ABDEL-MOHSEN, PH.D.
Assistant Professor
The Wistar Institute
3601 Spruce Street
Philadelphia, PA 19104
P (215) 898-3968 E mmohsen@Wistar.org

CEM.NKR.eGFP+ cells (fluorescently green cells) and co-culturing the mixture with NK cells isolated from HIV+ ART-suppressed individuals in the presence or absence of bNAb or conjugate. Data in **new Figure 7H-I** and **new Supplementary Figure 10** show the specificity and efficacy of the bNAb-sialidase conjugate in inducing NK-mediated killing of HIV+ cells.

In support of the reviewer's suggestion, we have also highlighted, in the Discussion section, the need to validate all of our findings using cells from HIV-infected ART-suppressed individuals in future studies. This addition to the Discussion section reads as follows:

"Next, for the NK cytotoxicity assays, we used certain viral isolates. In the future, it will be important to test other viral isolates, including transmitted/founder viruses. Furthermore, for the majority of these NK cytotoxicity assays, we used NK cells isolated from healthy donors (against HIV-infected cell lines or HIV-infected autologous CD4$^+$ T cells). However, validating our findings using cells from HIV-infected ART-suppressed individuals (from different clinical and demographic settings) will be important to ensure that HIV-mediated dysregulation of NK cell functions and phenotypes does not impact our findings."

3. *Furthermore, whether sialidase impact the effector functions of the anti-gp120 bNAbs directly as previously pointed out by the authors (PMID: 30707400) should be tested. Alternatively, blocking Fc receptors (CD16 on NK cells) in the cytotoxic assay would be important to further confirm the direct role of sialic acid in dampening NK cytotoxic responses.*

   Authors response: We thank the reviewer for this suggestion, and we have performed the suggested alternative experiment using Fc receptor blocker. Our new data (**new Figure 7G**) show that Fc block decreases the ability of bNAb to reduce levels of HIV-infected cells (as expected). However, the bNAb-sialidase conjugate was still able to enhance the ability of the bNAb to reduce levels of HIV-infected cells, despite the blocking of Fc receptors. Thus, these data suggest a direct role of the sialidase on the bNAb-conjugate ability to enhance NK cytotoxic responses.

Minor comments:

1. *Fig2A. The tSNE representation of concatenated Siglec-9+ CD56dim cells from HD, ART+ and viremic HIV-1 patients does not allow to identify neither clear clusters, nor differences between those 3 groups. Would a tSNE/UMAP representation of total CD56dim NK cells give better discrimination of NK clusters or differences between the groups?*

   Authors response: We thank the reviewer for this comment. We have tried UMAP analysis; however, the tSNE analysis still allowed for better separation. We have also elected to focus on Siglec-9$^+$ CD56^{dim} cells as this population is the focus of our manuscript.

2. *Fig2C. The choice of the illustrating dot plots is questionable, not displaying representative samples but instead the ones with the highest difference between Siglec-9+ and Siglec-9neg populations, possibly misleading the readers. The authors also focused on the description of Siglec-9+ vs. Siglec-9neg in HIV-1 patients; it would be important to describe the differences of NK phenotype between controls and patients irrespective of Siglec-9 expression (e.g. expression of CD38, CD161, NKp30, Siglec-7, TIGIT).*

   Authors response: We agree with the reviewer and thank him/her for this observation. More appropriate representative plots have now been selected for Figure 2C. However, given the focus of our manuscript, we focused on representative examples of HIV+ ART-suppressed donors rather than including plots for every study group.

3. *Fig3. The FlowSom analysis should be described in the material and methods section, with mention on how the number of clusters was pre-determined. Indeed, the tSNE representation (Fig3A) does not support the division in 8 different clusters, as only cells from cluster 5, 6 and 7 cluster together, while most cells are mixed, thus questioning the use of this analysis and the conclusions drawn from it.*

MOHAMED ABDEL-MOHSEN, PH.D.
Assistant Professor
The Wistar Institute
3601 Spruce Street
Philadelphia, PA 19104
P (215) 898-3968 E mmohsen@Wistar.org

Authors response: We agree with the reviewer and have now removed this section from the updated manuscript. We have also added to Figure 3 a new plot (new **Figure 3A**) showing a negative correlation between the frequency of Siglec-9^{+} CD56^{dim} NK cells and HIV plasma viral load in viremic individuals. This new analysis is consistent with the negative correlation we previously identified (new **Figure 3B**) between the Siglec-9^{+} CD56^{dim} NK cells sub-population and levels of cell-associated HIV DNA measured in the CD4^{+} T cells of HIV-infected ART-suppressed individuals.

4. *Fig3B. The MFI values represented in this heatmap do not correspond to the MFI reported in Fig2C. This heatmap does not show clear differences in marker expression between the different clusters, furrther questioning the cluster definition.*

   Authors response: We thank the reviewer for this comment, and we have now removed the clustering data from the updated manuscript.

5. *Figure 4. How the E:T ratio were set up for the different assays?*

   Authors response: Each of the assays (against cell lines or autologous CD4^{+} T cells) was optimized to select the E:T ratio optimal for the readout. For example, measuring NK (effector cells) degranulation usually requires a lower E:T ratio compared to measuring an impact on the target cells. By contrast, the E:T ratio in the killing assays against autologous HIV-infected CD4^{+} T cells needs to be higher than the ratio used in the killing assays against HIV-infected cell lines. We have now added the E:T ratios in the legend of each panel of each figure.

6. *Material and Methods: please describe the generation of in-house Siglec-9 Ab and how it was validated.*

   Authors response: We have now added a full section in the methods (and a **new supplementary Figure 11**) to describe the generation and characterization of this antibody.

7. *Fig S1B. Check the axis of the first dot plot.*

   Authors response: We thank the reviewer for noticing this oversight which we fixed. Note, this supplementary panel is now **Supplementary Figure 4**.

8. *Fig 1D. Check y axis legend.*

   Authors response: The axis label has been corrected.

9. *Fig 2A. Colour legend missing.*

   Authors response: The legend has been added.

10. *Line 89. Supplementary Figure 1B (instead of Supp. Figure 2).*

    Authors response: We thank the reviewer for noticing this oversight. We have fixed this mistake and this panel is now **Supplementary Figure 4**.

11. *Fig 6. Please indicate the E:T ratio. The statistics should be describe further, i.e. naming the test used for the multiple comparisons.*

    Authors response: We thank the reviewer for this comment. We have now added the E:T ratio to the legend of all figures. Also, we have now added Dr. Qin Liu (Professor of Biostatistics and Wistar's head of Biostatistics unit) to the authors of this manuscript. Dr. Liu reviewed all data analyses and performed modifications throughout the manuscript.

THE WISTAR INSTITUTE

MOHAMED ABDEL-MOHSEN, PH.D.
Assistant Professor
The Wistar Institute
3601 Spruce Street
Philadelphia, PA 19104
P (215) 898-3968 E mmohsen@Wistar.org

Reviewer #2

Reviewer general comment: *The study presented by the authors deals with a potential immune checkpoint molecule on NK cells and is thus of high relevance in the HIV research field and beyond that. The authors are very well known in the field of HIV for their outstanding expertise regarding glycosylation, inflammation and anti-HIV CD8+ T cell responses. Here they propose to apply their outstanding expertise and knowledge regarding glycosylation on NK cells. This study is original for several reasons. While Siglec-7 and other Siglecs have been studied on NK cells during HIV infection, the role of Siglec-9 has not been addressed. A negative correlation was observed between HIV DNA in CD4+ T cells and the percentage of Siglec-9+CD56dim NK cells during ART. Moreover, in the 2nd part of the presented work, the authors conjugated three bNabs to Sialidase and evaluated their impact on NK cell activity against HIV-infected cells. These conjugates selectively desialylated HIV-infected cells in vitro and enhanced NK cells' capacity to kill them.*

Authors response: We appreciate the reviewer's opinion of our findings and that he/she found them relevant and of interest. As detailed below, we have now addressed the concerns raised with new experiments and modifications throughout the manuscript.

Major comments:

1. *The gating strategies raise some questions. For example, on Figure 1A it seems that CD8+ cells have been excluded (dump CD3-CD8-DR-). However, a large proportion of the NK cells in human blood expresses CD8. Is this a typo or not a representative example of what has been done?*

   Authors response: We thank the reviewer for this comment. The reviewer is correct, and this was a typo. As shown in **Supplementary Figure 1**, we only excluded $CD19^+$, $CD14^+$, $CD3^+$, and dead cells. Then we focused on CD56, HLA-DR and CD16 for analyses. We have corrected the legend.

   Also, while in the past, many studies indeed excluded HLA-DR for NK cell gating, it is nowadays well known that NK cells can express HLA-DR. Of note, stimulated NK cells, for example through IL-2, express more often HLA-DR and can display enhanced activity against pathogens. For Siglec 7, it has been described that it becomes inhibitory on NK cells once the latter get activated. Thus, excluding HLA-DR from the gated NK cell population, might exclude a particularly interesting NK cell population to study in the analyses that used the gating strategy shown in Suppl. Fig1A (ie Figures 1,2,3A-C). Indeed, HLA-DR+ NK cells have been reported to be increased in HIV controllers (doi: 10.1073/pnas.1302090110) Another gating strategy, including the DR+ NK cells, might modify some of the conclusions of the work presented here.

   Authors response: We thank the reviewer for this valuable comment and suggestion. Indeed, many studies have reported the upregulation of HLA-DR on NK cells during HIV infection, not only in elite controllers but also in viremic individuals (Kuri-Cervantes L et al., *J Leukoc Biol.* 2014, PMID: 24399837). However, while doing the analyses, we found that most of the $CD3^-$ cells expressing HLA-DR fell on the $CD56^-$ population in all studied groups, as shown below.

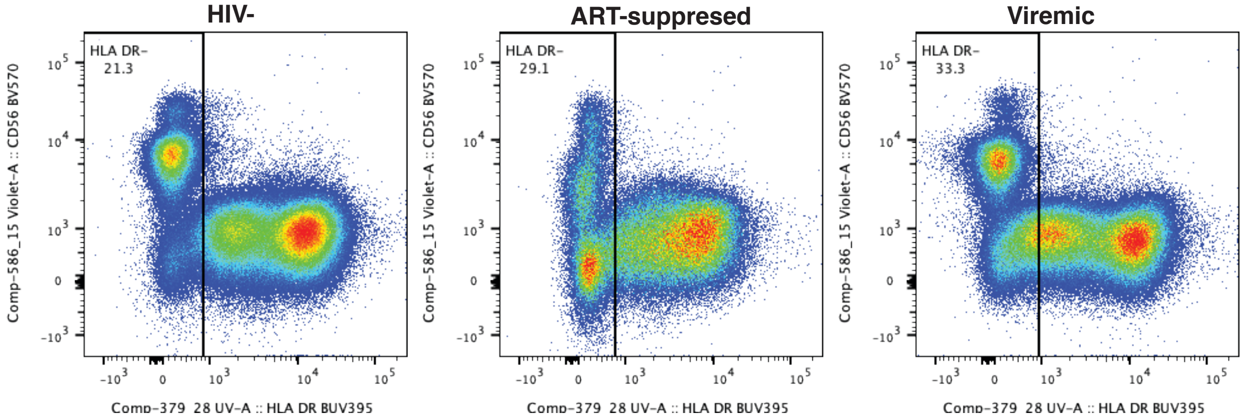

MOHAMED ABDEL-MOHSEN, PH.D.
Assistant Professor
The Wistar Institute
3601 Spruce Street
Philadelphia, PA 19104
P (215) 898-3968 E mmohsen@Wistar.org

Following the reviewer's suggestion, we have repeated the analysis of Siglec-9 expression on CD56^{dim} NK cells, including HLA-DR$^+$ cells (**new Supplementary Figure 2**). For this analysis, we characterized the frequency of Siglec-9$^+$ CD56^{dim} NK cells selecting from a Dump$^-$ (viable, CD14, CD19) CD3$^-$ gate. Then, focusing on CD56^{dim} cells, we gated on Siglec-9$^+$ cells. We excluded a small CD56^{low} population to avoid monocyte contamination (**new Supplementary Figure 2A**). In new Supplementary Figure 2B, we show the original gating strategy excluding HLA-DR+ NK cells and the downstream strategy. To confirm that we were not excluding a relevant NK cell subset, we also gated on the HLA-DR$^+$ population (**new Supplementary Figure 2C**) and explored the expression of Siglec-9. As shown, the cells within the HLA-DR$^+$ gate did not have an NK cell phenotype and were indicative of a monocyte population (CD56^{neg}, CD16^{bright}). Furthermore, almost none of these cells fell into the Siglec-9$^+$ gate used. Finally, we graphed the frequency of Siglec-9$^+$ CD56^{dim} NK cells following this new gating strategy (**new Supplementary Figure 2A**) and, although there was a discrete variation in the frequencies of Siglec-9$^+$ CD56^{dim} NK cells, the overall results were almost identical to those found when excluding HLA-DR$^+$ NK cells (Figure 1D). Given the focus of our analyses on 'cytotoxic' NK cells (CD56^{dim}) and the expression of Siglec molecules on monocytes, we elected to exclude HLA-DR from the subsequent analyses. This would ensure the exclusion of monocytes and allow for a cleaner analysis of NK cells. As shown in the graphs above and the new analysis performed, we concluded that the low frequency of HLA-DR$^+$ CD56$^+$ NK cells should not impact our overall results.

Figure 1B: Were the analyses performed in random order between donors from the distinct groups or was each group analysed separately? Were the antibody concentrations and/or gatings homogeneous in all experiments and between the groups?

Authors response: To avoid inter-experiment variation, the phenotyping experiment was performed on all samples simultaneously. All donor samples were thawed, stained, and processed simultaneously with a single antibody mix using similar cell numbers for each donor. All samples were then acquired as a single experiment and analyzed altogether. Donor groups were only separated once the data had been extracted to perform graphing and statistical analyses.

2. *The conclusions made for Figure 1 is a depletion of the Siglec-9+CD56dim NK cells in HIV infection. Other interpretations are also possible. Given the gating strategy, it is not excluded that they are not depleted but got activated and up-regulated DR, which would exclude them from the analysis. Only percentages are shown. Since the data are in blood, is it possible to show also the absolute numbers? A decrease in frequency can thus be a reflection of the increase of another population or also be due to a modulation of the phenotype (in chronic HBV infection, Siglec-9 expression is decreased).*

   Authors response: We thank the reviewer for this valuable comment. We agree with the reviewer and have now added these alternative explanations to the Discussion section as follows:

   "*During both cancer and HBV infection, the frequency of cytotoxic Siglec-9+ NK cells is reduced [24, 25]. Our data suggest a similar reduction in the frequency of Siglec-9+ CD56dim NK cells during HIV infection. However, there are several alternative explanations of this finding, including: 1) a change in the phenotype of these cells during HIV infection; 2) an increase in the frequency of other NK cell sub-populations; and 3) an increase in the migration ability of these cells from the blood to other body compartments.*"

3. *Fig.3.D. It could be interesting to have more detail about the subsets that correlate with HIV DNA during ART. The text says that total CD56dim Siglec9+ NK cells correlated with HIV DNA but that Siglec7 did not (data not shown). This is intriguing since the data show that siglec9+ NK cells are those that also express high levels of Siglec7 (Fig2B). Since Siglec7 is strongly expressed on the cluster with higher expression of activation markers, including Perforin, it could mean that the NK cell subpopulation that is decreased in HIV ART, is not the one with the higher functional activity. It could thus be interesting to also show the correlation with the other markers expressed by Siglec9+ NK cells or between the clusters they identified in the Figure 3 and the HIV DNA.*

MOHAMED ABDEL-MOHSEN, PH.D.
Assistant Professor
The Wistar Institute
3601 Spruce Street
Philadelphia, PA 19104
P (215) 898-3968 E mmohsen@Wistar.org

Authors response: This is an excellent point. However, perhaps our original text did not make it clear that the flow panel used in Figure 3D (now **Figure 3B**) is not the same as the flow panel used in Figures 1 and 2. The donors in Figure 3 are the donors with available CD4^{+} T cell-associated HIV DNA data. We agree with the reviewer that identifying a potential subpopulation of the Siglec-9^{+} CD56^{dim} NK cells that correlate best with CD4^{+} T cell-associated HIV DNA is an important task. We have now highlighted this point in the Discussion section (as shown in our response to point #7 below).

4. *Why cells from only three donors were tested in Fig.4C and Fig.4D, while in the Suppl.Fig.3, more donors were used.*

   Authors response: Evaluating all readouts from a donor's cells depended on the number of available cells recovered from this donor. All NK killing assays (both the assays against cell lines and the new assays using autologous target cells) require a large number of PBMCs, and there is considerable variability in the number of NK cells that can be successfully isolated from these PBMCs (depends on the % of NK cells in the blood of this donor and our isolation efficiency). On several occasions, we got a low number of PBMCs and/or isolated a low number of NK cells. On these occasions, we tested specific readouts and were not able to evaluate all possible readouts from these donors.

5. *The degranulation and IFN-g activity shown in these graphs are close to the threshold of statistical significance. Is this sufficient to conclude about the difference with regard to target cell specificity?*

   Authors response: We agree with the reviewer. However, the additional results from autologous NK killing assays, which are independently consistent with our original findings, support our conclusions. In the current version of the manuscript, each conclusion is supported with multiple experiments, usually using cells from multiple, and different donors. Thus, despite the small n per individual experiment, these experiments when considered collectively support one conclusion. For example, all experiments shown in Figures 7A-F, 7H-I, 8, and 9 are supporting one conclusion (namely that the bNAb-sialidase conjugate can induce NK cytotoxicity against HIV-infected cells more than the bNAb alone). These different experiments were done sepratly, using cells from different donors, and their results are consistent. Same is true for the different experiments in Figure 4, which all support one conclusion (that Siglec-9^{+} cells are more cytotoxic than Siglec-9^{-} cells against HIV infected cells) or Figure 5 (that Siglec-9 Ab can enhance NK cytotoxicity against HIV infected cells).

6. *Does the expression of Siglec-ligands on the K562 cells could explain the difference seen between the target cells?*

   Authors response: We believe the reviewer is correct and that the expression of the ligand could explain the difference between the target cells. However, our current data do not allow us to reach to this conclusion, and investigating this possibility, while important, is outside the scope of our current manuscript and we hope to investigate it in future work.

7. *Fig.5. This approach is not specifically directed against Siglec-9, which doesn't diminish the interest of the results. For the ability of the three conjugates to kill HXB2-infected CEM.NKR CCR5+ Luc+ cells, non-conjugated bNabs were used as negative controls. What about using bNabs conjugated to something else as negative control ? What would be the reduction of infection cells and cyotoxicity in that case?*

   Authors response: We agree with the reviewer that the bNAb-sialidase approach is not specific to Siglec-9 and should prevent all other Siglec interactions. We believe that this feature could actually make this approach more compelling. Using bNAbs conjugated to non-specific enzyme (as a negative control instead of the non-conjugated bNAbs) is an excellent idea; however, implementing this idea could be challenging. We would have to ensure that this other, non-specific enzyme: 1) has no other direct or indirect effects on the target cells; and 2) has the same size and physical properties as the sialidase. For these reasons, we elected to use non-conjugated bNAbs as controls; however, we have highlighted the need for more controls in the Discussion section, as suggested by the reviewer.

MOHAMED ABDEL-MOHSEN, PH.D.
Assistant Professor
The Wistar Institute
3601 Spruce Street
Philadelphia, PA 19104
P (215) 898-3968 E mmohsen@Wistar.org

"Regarding the experiments in Figures 7-9, we used the bNAbs alone as controls to examine the impact of bNAb-sialidase conjugates on NK cytotoxicity; it will be important, in the future, to examine bNAbs conjugated to non-specific enzymes.''

8. *Fig.5: A confirmation of the functional assays using primary cells and primary HIV isolates would support the data.*

Authors response: We thank the reviewer for this valuable suggestion. We have performed several new experiments using autologous NK and HIV-infected primary cells to support many of our original findings. First, in the **new Figure 4E-F**, we show that Siglec-9^{+} CD56^{dim} NK cells are more cytotoxic against autologous HIV-infected CD4^{+} T cells than are Siglec-9^{-} CD56^{dim} NK cells from the same donors. Second, we show that Siglec-9^{+} blocking antibody selectively increases Siglec-9^{+} CD56^{dim} NK degranulation (**new Figure 5D-E**) and decreases levels of HIV infection (**new Figure 5D, F and new Supplementary Figure 7**) in autologous HIV-infected CD4^{+} T cells. Finally, we show that the bNAb-Sialidase conjugate increases NK degranulation (**new Figure 8**) and decreases HIV infection (**new Figure 9**) in an autologous killing assay compared to the bNAb alone.

Each of these experiments was repeated independently three times using cells from different donors, and each repeat was done in multiple technical replicates.

We again thank the reviewer for raising this critical point, as we believe the addition of these new autologous experiments added more depth and sophistication to our manuscript.

Regarding the usage of primary HIV isolates, we agree with the reviewer; however, given the nature of the autologous killing assays (requires a large number of freshly acquired primary cells, time-consuming, and requires high infection rates of the CD4^{+} T cells), we had to optimize our autologous NK killing assays using IIIB-HIV. However, we have added this caveat to the Discussion section and highlighted the need to test other viral isolates, including transmitted/founder viruses.

"Next, for the NK cytotoxicity assays, we used certain viral isolates. In the future, it will be important to test other viral isolates, including transmitted/founder viruses. Furthermore, for the majority of these NK cytotoxicity assays, we used NK cells isolated from healthy donors (against HIV-infected cell lines or HIV-infected autologous CD4^{+} T cells). However, validating our findings using cells from HIV-infected ART-suppressed individuals (from different clinical and demographic settings) will be important to ensure that HIV-mediated dysregulation of NK cell functions and phenotypes does not impact our findings."

9. *The Siglec-9+ CD56dim NK cells are, in concordance with other studies in the literature, corresponding to a more activated, cytotoxic and mature phenotype (Figs 1-3). To which NK cell population described in other HIV/SIV studies do these Siglec-9+ CD56dim NK cells probably correspond the most? In other words, are the dynamics of these NK cells with regard to activation and maturation state, and association with viral load, showing similarities with other mature NK cell populations described in other studies on HIV/SIV infections ? This could be developed in the discussion part.*

Authors response: We thank the reviewer for his/her insightful question and valuable suggestion to expand our discussion section. We have added the following text to the Discussion to address the reviewer's comment.

"Little is known about Siglec-9 expression on NK cells in general, and no description of Siglec-9 phenotype or function on NK cells has been reported in the context of HIV infection. We performed both phenotypic and functional analyses of Siglec-9^{+} NK cells during HIV infection. Our data show that NK cells expressing Siglec-9 bear a more activated phenotype (higher CD38) and a stronger functional profile (CD16, DNAM-1, Perforin, and NKp30) than Siglec-9^{-} NK cells. While this suggests that Siglec-9+ NK cells represent a more mature NK subset, we found no difference in CD57 expression between Siglec-9^{+} and Siglec-9^{-} NK cells in people living with HIV (PLWH) from our study. However, previous

NCI Cancer Center
A Cancer Center Designated by the National Cancer Institute

MOHAMED ABDEL-MOHSEN, PH.D.
Assistant Professor
The Wistar Institute
3601 Spruce Street
Philadelphia, PA 19104
P (215) 898-3968 E mmohsen@Wistar.org

work from other groups showed that Siglec-9$^+$ NK cells in control donors exhibit a more mature phenotype (higher CD57, CD69, and KLRG1) [24]. Previous studies have shown that Siglec-7$^+$ CD56$^+$ NK cells also have a stronger functional profile than Siglec-7$^-$ CD56$^-$ NK and Siglec-7$^-$ CD56$^+$ NK cells [17]. More recently, it was reported that the frequency of the CD11b$^+$ CD57$^-$ CD161$^+$ Siglec-7$^+$ subpopulation of CD56dim CD16+ NK cells is lower in viremic HIV-infected individuals, correlates negatively with levels of HIV DNA, and exhibits higher cytokine and degranulation responses against K562 target cells [52]. Our data show that Siglec-7 is expressed at higher levels on Siglec-9$^+$ CD56^{dim} NK cells. Future studies are needed to perform an in-depth phenotypic analysis of the Siglec-9$^+$ CD56^{dim} NK cells subpopulation and determine its potential overlap with the CD11b$^+$ CD57$^-$ CD161$^+$ Siglec-7$^+$ subpopulation. Other NK subpopulations have also been identified as representing a mature and differentiated phenotype during HIV/SIV infection, such as the CXCR5$^+$ [53] and NKG2A^{Low} [54] NK cells in secondary lymphoid tissues of SIV-infected African green monkeys (AGM). Future work will also need to address whether Siglec-9 is differentially expressed on these subpopulations in blood and in tissues, the main site for HIV persistence."

17. Brunetta E, Fogli M, Varchetta S, Bozzo L, Hudspeth KL, Marcenaro E, et al. The decreased expression of Siglec-7 represents an early marker of dysfunctional natural killer-cell subsets associated with high levels of HIV-1 viremia. Blood. 2009;114(18):3822-30. Epub 2009/08/28. doi: 10.1182/blood-2009-06-226332. PubMed PMID: 19710502; PubMed Central PMCID: PMCPMC2773483.

24. Jandus C, Boligan KF, Chijioke O, Liu H, Dahlhaus M, Demoulins T, et al. Interactions between Siglec-7/9 receptors and ligands influence NK cell-dependent tumor immunosurveillance. J Clin Invest. 2014;124(4):1810-20. Epub 2014/02/27. doi: 10.1172/JCI65899. PubMed PMID: 24569453; PubMed Central PMCID: PMCPMC3973073.

52. Pohlmeyer CW, Gonzalez VD, Irrinki A, Ramirez RN, Li L, Mulato A, et al. Identification of NK Cell Subpopulations That Differentiate HIV-Infected Subject Cohorts with Diverse Levels of Virus Control. J Virol. 2019;93(7). Epub 2019/02/01. doi: 10.1128/JVI.01790-18. PubMed PMID: 30700608; PubMed Central PMCID: PMCPMC6430550.

53. Huot N, Jacquelin B, Garcia-Tellez T, Rascle P, Ploquin MJ, Madec Y, et al. Natural killer cells migrate into and control simian immunodeficiency virus replication in lymph node follicles in African green monkeys. Nat Med. 2017;23(11):1277-86. Epub 2017/10/17. doi: 10.1038/nm.4421. PubMed PMID: 29035370; PubMed Central PMCID: PMCPMC6362838.

54. Huot N, Rascle P, Petitdemange C, Contreras V, Sturzel CM, Baquero E, et al. SIV-induced terminally differentiated adaptive NK cells in lymph nodes associated with enhanced MHC-E restricted activity. Nat Commun. 2021;12(1):1282. Epub 2021/02/26. doi: 10.1038/s41467-021-21402-1. PubMed PMID: 33627642; PubMed Central PMCID: PMCPMC7904927.

Minor comments:

1. *Line 79, line 82-83: Clusters 5 and 7 are decreased in viremic, not in ART (line 79).*

   Authors response: Based on comments from Reviewers 1 and 3, we have now removed this section from the updated manuscript.

2. *Fig 3: The sum of clusters was not close 100%. This could probably easily be explained by many little subpopulations that are not part of any cluster. Add more explanations on Fig.3A.*

   Authors response: In response to the Reviewers' comments, Figure 3A-C have been removed.

3. *The number of donors should be mentioned for each figure (for ex Fig 4B, the text says "several" donors, line 100).*

   Authors response: We thank the reviewer for this suggestion and have now listed the n to the legend of every panel of every figure.

4. *Fig. 4D on line 115 probably means Figure 4B.*

   Authors response: We apologize for this oversight and have fixed this typo.

THE WISTAR INSTITUTE

MOHAMED ABDEL-MOHSEN, PH.D.
Assistant Professor
The Wistar Institute
3601 Spruce Street
Philadelphia, PA 19104
P (215) 898-3968 E mmohsen@Wistar.org

Reviewer #3

Reviewer general comment: Adeniji et al. describe a novel method to improve the ability of NK cells to target HIV-infected cells by releasing the brakes induced by Siglec-9 expression on a subset of highly cytotoxic NK cells. This manuscript begins by demonstrating that Siglec-9 is expressed on a subset of NK cells with high expression of activating NK cell receptors capable of enhancing NK cell cytotoxicity. They show an impressive inverse correlation between the frequency of Siglec-9+ NK cells and the level of the HIV reservoir, suggesting that Siglec-9+ NK cells could play a role in limiting reservoir size. In vitro, they demonstrated that Siglec-9+ NK cells had high cytotoxicity, but that Siglec-9, as an inhibitor receptor, restrains their cytotoxic function. In a very innovative approach, they devised an approach to harness NK cell activity by selectively disrupting Siglec/sialoglycan interactions by conjugating sialidase to HIV-specific bNAbs, revealing that this could enhanced the ability of NK cells to kill HIV-infected cells. This paper breaks new ground by defining a previously unappreciated pathway to enhance the ability of NK cells to kill HIV-infected cells. This is a particularly important area of investigation as various "shock-and-kill" strategies employing NK cells for the kill gain traction. This could therefore define a high-impact area, particularly given recent promising results in targeting Siglecs in the setting of autoimmunity and cancer as proof of principle for in vivo effects of this pathway. The paper takes a creative approach to a neglected area, and the approach of harnessing bNAbs to selective di-sialate cells is creative and interesting. However, there are several concerns that should be addressed to assure the specificity and impact of the findings. The most critical concern is that the authors do not demonstrate that primary HIV-infected CD4+ T cells express sufficient sialoglycans to have the siglec/sialoglycan axis play a role in suppressing NK cell killing in the real world. If this is just an observation in cultured cells that express high levels, then it is of limited utility. Using more relevant HIV strains (e.g. T/F strains rather than lab-adapted) and infected cells (primary CD4+ T cells) is imperative to fully establish this as an important pathway. Additional demonstration that the observed effects are not due, at least in part, to monocytes, should also be addressed. Finally, the very small number of replicates (and resulting inapproapriate statistics) is concerning and undermines confidence for many of the major findings in Figures 4-6.

Authors response: We appreciate the reviewer's opinion of our findings and that he/she found them to break new ground and be of a high impact. As detailed below, we have now addressed the concerns raised with new experiments and modifications throughout the manuscript.

Major comments:

1. *A major concern about the broader applicability of the results comes from the fact that the experiments were largely done in an experimental system using HuT78 cells that have high levels of Siglec-9 ligands compared to primary T cells. Without demonstration that Siglec-9 engagement dampens NK cell responses to HIV-infected primary CD4+ T cells, it is unclear whether this observation is relevant/important to HIV pathogenesis.*

   Authors response: We thank the reviewer for this valuable suggestion. We have performed several new experiments using autologous NK and HIV-infected primary cells to support many of our original findings. First, in the **new Figure 4E-F**, we show that Siglec-9^{+} CD56^{dim} NK cells are more cytotoxic against autologous HIV-infected CD4^{+} T cells than are Siglec-9^{-} CD56^{dim} NK cells from the same donors. Second, we show that Siglec-9^{+} blocking antibody selectively increases Siglec-9^{+} CD56^{dim} NK degranulation (**new Figure 5D-E**) and decreases levels of HIV infection (**new Figure 5D, F and new Supplementary Figure 7**) in autologous HIV-infected CD4^{+} T cells. Finally, we show that the bNAb-Sialidase conjugate increases NK degranulation (**new Figure 8**) and decreases HIV infection (**new Figure 9**) in an autologous killing assay compared to the bNAb alone.

   Each of these experiments was repeated independently three times using cells from different donors, and each repeat was done in multiple technical replicates.

   We again thank the reviewer for raising this critical point, as we believe the addition of these new autologous experiments added more depth and sophistication to our manuscript.

MOHAMED ABDEL-MOHSEN, PH.D.
Assistant Professor
The Wistar Institute
3601 Spruce Street
Philadelphia, PA 19104
P (215) 898-3968 E mmohsen@Wistar.org

2. *Another concern is the selection of viruses used: SF2 (dual tropic) and other lab-adapted strains. In this era where a variety of T/F and other primary viruses are readily available, it is important to demonstrate that this is relevant in 'real-world' viruses.*

   Authors response: We agree with the reviewer; however, given the nature of the autologous killing assays (requires a large number of freshly acquired primary cells, time-consuming, and requires high infection rates of the $CD4^+$ T cells), we had to optimize our autologous NK killing assays using HIV-IIIB. However, we have added this caveat to the Discussion section and highlighted the need to test other virus isolates, including transmitted/founder viruses.

   "Next, for the NK cytotoxicity assays, we used certain viral isolates. In the future, it will be important to test other viral isolates, including transmitted/founder viruses. Furthermore, for the majority of these NK cytotoxicity assays, we used NK cells isolated from healthy donors (against HIV-infected cell lines or HIV-infected autologous $CD4^+$ T cells). However, validating our findings using cells from HIV-infected ART-suppressed individuals (from different clinical and demographic settings) will be important to ensure that HIV-mediated dysregulation of NK cell functions and phenotypes does not impact our findings."

3. *Better explanation of the gating scheme for the characterization of the NK cell population is needed for Figures 1 and 2, and possible adjustments may be required. The overarching concern is this: many times monocytes can appear in NK cell gates because monocytes, particularly after activation, can upregulate CD56 (for example, Milush et al. Blood 2009 PMID 19805616 and Krasselt et al 2013 PMID 24286519). Further, as Siglec-9 is expressed on a relatively small subset of NK cells, but is somewhat universally high on monocytes, this raises a real concern as to whether some of the Siglec-9+ NK cells could actually be monocytes. This seems unlikely in light of the co-expression of NKp30 and other more NK-cell specific markers, but many times these markers can be somewhat fluid in their definition, and care needs to be made to assure that monocytes are not explaining the results. For instance, some assays that were thought to be specific to NK cells for ADCC might actually represent activity of monocytes, and this is a very important consideration (Kramski et al., 2012 PMID 22841577). Specific concerns/questions about the gating include:*

   a. *Based on the Milush paper above, it would be ideal to include CD7 in the gating strategy as this should more definitively exclude monocytes as monocytes do not express CD7. Expression of CD7 can be more variable on CD56bright NK cells, but as this study focuses on CD56dim cells that should not present a problem. It is understandable if samples from the same cohort cannot be restained, but at least some healthy controls should be stained with a panel inclusive of CD7 to assure that inclusion of this gate does not eliminate the Siglec-9 expressing cells, because, in fact, some are monocytes. With regard to this it is important to note that not only can monocytes upregulate CD56, but that they can also downregulate HLA-DR (Abeles et al, Cytometry A 202 PMID 22837127). In particular, looking at the HLA-DR gate, it looks like there may be a low, mid, and high, and the gate may cut through the mid, thus including potential monocytes in the gating strategy.*

   Authors response: We followed the reviewer's suggestion and tested whether $Siglec\text{-}9^+$ $CD56^{dim}$ NK cells express CD7. Using cells from a healthy donor (**new Supplementary Figure 3**), we showed that almost all $Siglec\text{-}9^+$ $CD56^{dim}$ NK cells are indeed expressing CD7, decreasing the possibility that they are monocytes.

   b. *Unclear why CD8 was a negative gate, as a subset of NK cells do express CD8, and in fact, increase in CD8+ NK cells is associated with lower HIV disease progression (Ahmad et al 2014 PMID 25122796). By gating out this population of highly mature and responsive NK cells, the authors could have enriched for a more rare subset of responding cells with Siglec-9, if they had, in fact, removed most of the HIV-responsive cells.*

MOHAMED ABDEL-MOHSEN, PH.D.
Assistant Professor
The Wistar Institute
3601 Spruce Street
Philadelphia, PA 19104
P (215) 898-3968 E mmohsen@Wistar.org

Authors response: We thank the reviewer for this comment and apologize for this typo. CD8$^+$ was not excluded. As shown in **Supplementary Figure 1**, we only excluded CD19$^+$, CD14$^+$, CD3$^+$, and dead cells. Then we focused on CD56, HLA-DR and CD16 for analyses. We have now fixed the legend.

Minor comments:

1. *It would be helpful throughout the manuscript if the n was listed in the figure legend.*

   Authors response: We thank the reviewer for this suggestion and have now listed the n to the legend of every figure.

2. *Assuming the caveats above are taken care of (ie that monocytes in the NK cell gating scheme are not driving results), the inverse relationship between the frequency of Siglec-9+ NK cells and HIV reservoir is impressive. Frankly, it is unclear what additional information, if any, is derived from the clustering analysis in Figures 3A-C as they do not significantly add to the conclusions.*

   Authors response: We agree with the reviewer and have now removed the clustering analysis from the updated manuscript.

3. *Overall, the statistics in this paper are sorely lacking. In many cases, a parametric test is used with very small n (t-tests used repeatedly with n=3). Parametric tests require normally distributed data, but it is impossible to ascertain normality of data with such a small n.*

   Authors response: We have now added Dr. Qin Liu (Professor of Biostatistics and Wistar's head of Biostatistics unit) to the authors of this manuscript. Dr. Liu reviewed all data analyses and performed modifications throughout the manuscript. Due to the limited number of donors and the time-consuming experimental procedure, we were only able to carry out each experiment with 3-4 independent biological replicates (however, each of these biological replicates was performed in multiple technical replicates). With this sample size, it is not possible to test the data normality. It is important to note that in the current version of the manuscript, each conclusion is supported with multiple experiments, usually using cells from multiple, and different donors. Thus, despite the small n per individual experiment, these experiments when considered collectively support one conclusion. For example, all experiments shown in Figures 7A-F, 7H-I, 8, and 9 are supporting one conclusion (namely that the bNAb-sialidase conjugate can induce NK cytotoxicity against HIV-infected cells more than the bNAb alone). These different experiments were done sepratly, using cells from different donors, and their results are consistent. Same is true for the different experiments in Figure 4, which all support one conclusion (that Siglec-9$^+$ cells are more cytotoxic than Siglec-9$^-$ cells against HIV infected cells) or Figure 5 (that Siglec-9 Ab can enhance NK cytotoxicity against HIV infected cells).

4. *The data presented in Figures 4-6 present a compelling and novel story that Siglec-9+ NK cells are enhanced in killing HIV-infected cells and that this activity can be further enhanced by blocking the ability to Siglec-9 to interact with its ligands and dampen NK cell activation. The fact that purified NK cells were used lessens the concerns raised above about contaminating monocytes (yet not fully given the explanation above in #1). However, there are a few concerns that dampen confidence in these findings. First, related to the statistics above, most experiments were done with only 3 donors (except Figure 6D-F which used 4, likely because one of the four doesn't show the desire effect?). As these experiments use healthy donor NK cells, this seems an unreasonably small size at it seems reasonable to ask for a few more replicates to assure that this is a reproducible effect given heterogeneity in NK cell populations between donors.*

   Authors response: We have now added Dr. Qin Liu (Professor of Biostatistics and Wistar's head of Biostatistics unit) to the authors of this manuscript. Dr. Lui reviewed all data analyses and performed modifications throughout the manuscript. We have also supported all of the findings in Figures 4-6 with additional autologous experiments (Figures 4E-F, 5D-F, 7H-I, 8, and 9; all new experiments done using different donors than the cell line experiments).

MOHAMED ABDEL-MOHSEN, PH.D.
Assistant Professor
The Wistar Institute
3601 Spruce Street
Philadelphia, PA 19104
P (215) 898-3968 E mmohsen@Wistar.org

These autologous killing experiments require a large number of cells to be able to isolate $CD4^+$ T cells (to infect them) and NK cells (when applicable) and perform all proper controls in multiple replicates. Each of these experiments requires, on average, ~300 million PBMCs (sometimes to measure only a single outcome, such as NK degranulation or p24 expression as the E:T ratio needed for each readout is different). This number of PBMCs requires ~300 ml of blood from each donor. More importantly, all of these autologous experiments required the usage of freshly acquired cells (as cryopreservation can impair NK cytotoxicity; Mark et al., *Nature Communications*, 2020 PMID: 33067467). Importantly, we did not omit any results. The NK killing assays (both the assays against cell lines and the new assays using autologous target cells) require a large number of freshly isolated PBMCs, and there is considerable variability in the number of NK cells that can be successfully isolated from these PBMCs (depends on the % of NK cells in the blood of this donor). On several occasions, we got a low number of freshly isolated PBMCs and/or isolated a low number of NK cells, in in such situations we only performed limited assays.

With the addition of several autologous assays that we performed for the new version of this manuscript, each observation is now supported by several experiments (using both cell lines and primary cells; each of these experiments was done in both biological and technical replicates and using different donors), giving us more confidence in our observations. However, we have added to the Discussion section the need to test our findings using cells from HIV+ individuals in different settings.

"Next, for the NK cytotoxicity assays, we used certain viral isolates. In the future, it will be important to test other viral isolates, including transmitted/founder viruses. Furthermore, for the majority of these NK cytotoxicity assays, we used NK cells isolated from healthy donors (against HIV-infected cell lines or HIV-infected autologous $CD4^+$ T cells). However, validating our findings using cells from HIV-infected ART-suppressed individuals (from different clinical and demographic settings) will be important to ensure that HIV-mediated dysregulation of NK cell functions and phenotypes does not impact our findings."

5. *The quite interesting results in Figure 4D-F rely on an antibody to block Siglec-9. Validation of this antibody should be provided either through reference in the supplement. This could be done by evaluating SHP-1 phosphorylation.*

Authors response: We thank the reviewer for this comment. We have now added detailed information, in the methods section, on the generation and characterization of this antibody. As mentioned in this section, hybridomas of 1152 Ab candidates were generated and screened using ELISA and western blotting to select antibody that binds selectively to Siglec-9 (**new supplementary Figure 11**). In addition, our data in **Figure 5A** show that the Siglec-9 blocking Ab has no effects on Siglec-$9^{depleted}$ NK cells (compared to total NK cells). Furthermore, data in **new Figure 5E** show that this antibody selectively induces the degranulation of Siglec-9^+ $CD56^{dim}$ NK cells compared to Siglec-9^- $CD56^{dim}$ NK cells in autologous assays. These data suggest the selectivity of this antibody towards SIglec-9.
